# Melatonin Is Neuroprotective in *Escherichia coli* Meningitis Depending on Intestinal Microbiota

**DOI:** 10.3390/ijms24010298

**Published:** 2022-12-24

**Authors:** Dong Zhang, Shu Xu, Hucong Wu, Jiaqi Liu, Yiting Wang, Guoqiang Zhu

**Affiliations:** 1 College of Veterinary Medicine, Yangzhou University, Yangzhou 225009, China; 2 Joint Laboratory of International Cooperation on Prevention and Control Technology of Important Animal Diseases and Zoonoses of Jiangsu Higher Education Institutions, Jiangsu Co-Innovation Center for Prevention and Control of Important Animal Infectious Diseases and Zoonoses, Yangzhou 225009, China; 3 Joint International Research Laboratory of Agriculture and Agri-Product Safety, The Ministry of Education of China, Yangzhou University, Yangzhou 225009, China

**Keywords:** bacterial meningitis, APEC TW-XM, melatonin, blood–brain barrier, inflammation, intestinal microbiota

## Abstract

Avian meningitis *Escherichia coli* (*E. coli*) can cause acute bacterial meningitis which threatens poultry health, causes great economic losses in the poultry industry, and has recently been speculated as a potential zoonotic pathogen. Melatonin can counteract bacterial meningitis-induced disruption of the blood–brain barrier (BBB), neuroinflammation, and reduce mortality. There are increasing data showing that melatonin’s beneficial effects on bacterial meningitis are associated with intestinal microbiota. In this study, our data showed that melatonin alleviated neurological symptoms, enhanced survival rate, protected the integrity of the BBB, reduced the bacterial load in various tissues and blood, and inhibited inflammation and neutrophil infiltration of brain tissue in an APEC TW-XM-meningitis mice model. The results of 16S rRNA showed that melatonin pretreatment significantly maintained the composition of intestinal microbiota in APEC-meningitis mice. The abundance and diversity of intestinal microbiota were disturbed in APEC TW-XM-meningitis mice, with a decreased ratio of *Firmicutes* to *Bacteroides* and an increased the abundance of *Proteobacteria*. Melatonin pretreatment could significantly improve the composition and abundance of harmful bacteria and alleviate the decreased abundance of beneficial bacteria. Importantly, melatonin failed to affect the meningitis neurologic symptoms caused by APEC TW-XM infection in antibiotic-pretreated mice. In conclusion, the results suggest that melatonin can effectively prevent meningitis induced by APEC TW-XM infection in mice, depending on the intestinal microbiota. This finding is helpful to further explore the specific target mechanism of melatonin-mediated intestinal microbiota in the prevention of and protection against *Escherichia coli* meningitis.

## 1. Introduction

Avian pathogenic *Escherichia coli* (APEC) are an important extraintestinal pathogenic *Escherichia coli* (ExPEC) [1]. Due to different serotypes, it can cause different diseases, such as diarrhea, pneumonia, endocarditis, septicemia, and meningitis in poultry. In addition, researchers have also found that meningitis-causing APEC and neonatal meningitis *Escherichia coli* (NMEC) have similar genome structures and the same animal model; there are also high similarities in genetic evolution and ecological distribution [2,3]. These evidence suggest that meningitis-causing APEC has a potential risk of zoonosis, which not only causes huge economic losses in the poultry industry, but also threatens human health. APEC TW-XM can infect Muscovy ducks, chickens, and mice and cause severe acute septicemia and meningitis, which show obvious meningitis neurological symptoms with a high probability of acute death [4]. At the same time, it was also found that the host produced severe systemic inflammation, systemic infection, and the destruction of the blood–brain barrier (BBB). In addition, the BBB is key to establishing and maintaining homeostasis in the brain. Moreover, it was found that, clinically, the symptoms of bacterial meningitis infection include anorexia, vomiting, and diarrhea, which reflect the changes in the intestinal homeostasis and affect the development of the disease [5]. For example, *Listeria monocytogenes* infection can change the intestinal microbiota of the host, which induces an increase in the abundance of *Alloprevotella*, *Allobaculum*, and *Streptococcus* in the intestinal tract, which destroys the integrity of the intestinal barrier and enters the abdominal blood circulation to promote severe septicemia or meningitis [6]. At present, the use of antibiotics to treat *Escherichia coli* meningitis can reduce mortality, but it leads to the emergence of more drug-resistant *Escherichia coli* and increases the difficulty of treatment.

Melatonin (MT), originally found to be an indole neuroendocrine hormone, is synthesized and secreted by the pineal gland of the brain [7,8]. The amphiphilic melatonin can easily cross the BBB to enter the central nervous system and cerebrospinal fluid [9,10]. This is particularly important for the effective prevention and treatment of central nervous system diseases after exogenous melatonin supplementation. It is well known that melatonin regulates circadian rhythm, sleep, and reproduction. Subsequently, a large number of studies have shown that melatonin has many other important functions, such as antibacterial, antioxidant, anti-inflammatory, and antiapoptosis functions, as well as the regulation of the immune system and intestinal microbiota [11,12,13]. Clinically, the preventive or therapeutic use of melatonin should be alternatively considered. Therefore, as a natural small molecular substance, melatonin is relatively safe compared to antibiotics. It has a low risk of side effects, so it can be used as a candidate for alternative antidrugs [14]. At present, it has been reported that melatonin had the beneficial effects of on protecting the integrity of the BBB, inhibiting neuronal and glial damage in various central nervous system disease models [11,12,13]. It was reported that 100 mg/kg of melatonin as a therapeutic agent decreased proinflammatory cytokine levels and the number of apoptotic neurons in *Klebsiella pneumoniae*-infected rats [12]. Recently, it is worth noting that melatonin can influence intestinal microbiota [15,16,17,18]. Melatonin supplements can reshape the composition of intestinal microbiota, resulting in the alleviation of weanling-induced stress [15]. Meanwhile, melatonin was also demonstrated to reduce the bacterial load of enterotoxigenic *Escherichia coli* (ETEC) in weanling mice, depending on the intestinal microbiota [15].

Although the effect of melatonin on intestinal microbiota has been noticed, until recently few investigations have focused on the beneficial preventive effects of melatonin on *Escherichia coli* meningitis by regulating intestinal microbiota. Therefore, 3-week-old ICR mice were injected intraperitoneally with different concentrations of melatonin, and then infected with meningitis strain APEC TW-XM after pretreatment for one week in this study. The incidence and death of *Escherichia coli* meningitis in mice were observed, and the effects of melatonin supplements on APEC TW-XM pathogenicity, the BBB integrity, inflammation, intestinal microbiota were explored.

## 2. Results

### 2.1. Melatonin Supplementation Decreases APEC TW-XM Pathogenicity in ICR Mice

**Figure 1:**
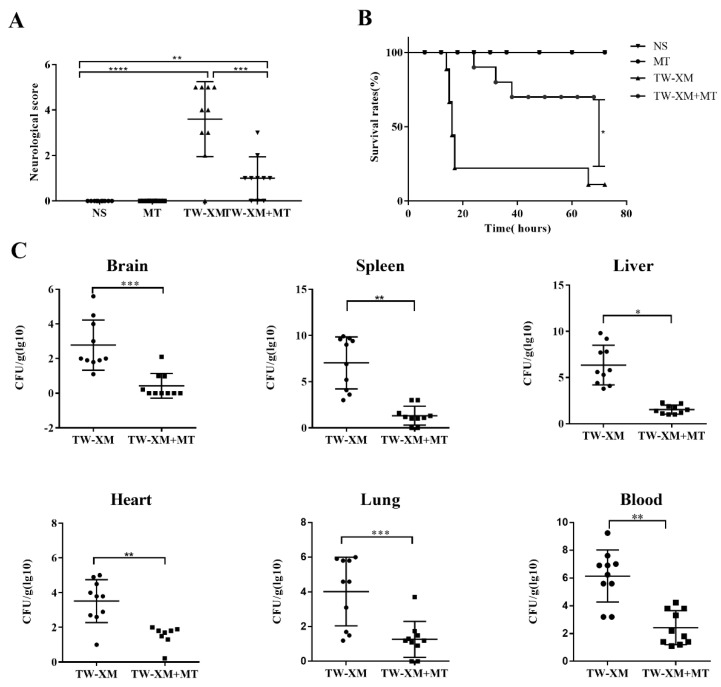
The effect of melatonin on neurological symptoms, survival rates, and bacterial counts of tissues and blood in TW−XM−infected ICR mice. (**A**) The neurological symptom scores of ICR mice in different groups were analyzed at 12 h post infection. (**B**) The survival rates of ICR mice in different groups were analyzed. (**C**) The loads of TW−XM in brain, spleen, liver, heart, lungs, and blood were analyzed in the TW−XM group and the TW-XM+MT group at 12 h post infection. Each point represents one mouse. All data were determined by one-way ANOVA and expressed as means ± SEM. * *p* < 0.05, ** *p* < 0.01, *** *p* < 0.001, **** *p* < 0.0001.

To determine the preventive effects of melatonin on TW-XM pathogenicity in ICR mice, three-week-old male ICR mice were intraperitoneally injected either with melatonin (10 mg/kg/day; 30 mg/kg/day; 60 mg/kg/day) [12,15,19,20] or NS for seven consecutive days before TW-XM infection. As shown in Supplementary Figure S1 and Figure 1A, 8 h after infection, mice in the TW-XM and TW-XM+MT_10 mg/kg_ groups began to develop neurological symptoms of meningitis, including mental malaise, anorexia, increased eye secretions, and unformed feces. After about 12 h, the mice developed convulsions, neck stiffness, and, finally, frequently died of angular arch reversal. The mice in the TW-XM+MT_30 mg/kg_ group and the TW-XM + MT_60 mg/kg_ group still showed lethargy, less food intake, partial eye secretion to a certain extent, and a few phenomena such as unformed feces, convulsions, and neck stiffness, which were significantly lower than those in the TW-XM group (*p* < 0.05). As shown in Supplementary Figure S2 and Figure 1B, the survival rates of the TW-XM group and the TW-XM + MT_10 mg/kg_ group were significantly lower than that of the TW-XM + MT30mg/kg group and the TW-XM + MT_60 mg/kg_ group, that is, the mortality rates of the TW-XM group, TW-XM + MT_10 mg/kg_ group, TW-XM + MT_30 mg/kg_ group, and TW-XM+MT_60 mg/kg_ group were 90%, 90%, 30%, and 40%, respectively. The above results show that the pretreatment of melatonin at the doses of 30 mg/kg and 60 mg/kg can significantly improve the survival rates of mice and effectively prevent the occurrence of meningitis in mice infected with APEC TW-XM. Therefore, melatonin at the dose of 30 mg/kg was chosen as the concentration to prevent the occurrence and development of meningitis.

Then, bacterial counts in the brain, spleen, liver, heart, lungs, and blood were, respectively, analyzed, and the results showed that the bacterial counts in the above tissues and blood were more strongly reduced when compared with the TW-XM group (Figure 1C). Collectively, these data demonstrated that melatonin could significantly reduce APEC TW-XM pathogenicity in ICR mice.

### 2.2. Melatonin Supplementation Protects the BBB Integrity in APEC TW-XM-Infected Mice

**Figure 2:**
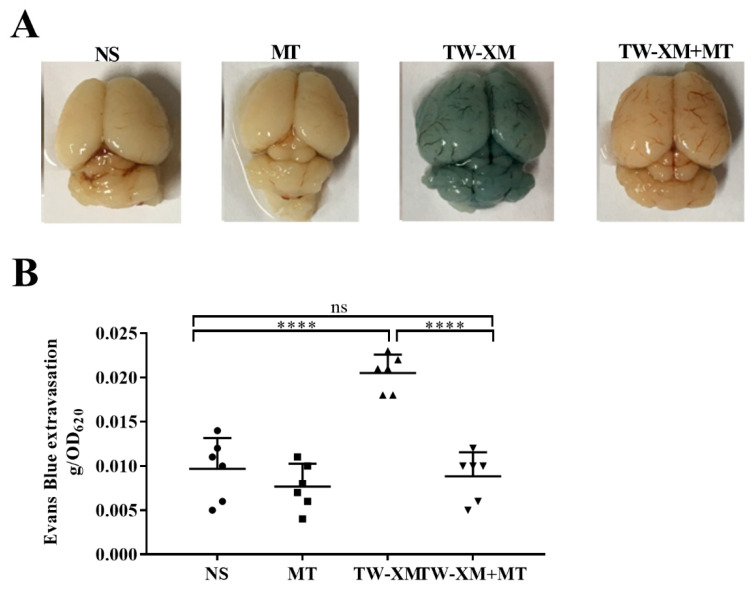
The effect of melatonin on the BBB integrity in APEC TW−XM−infected mice. (**A**,**B**) The BBB integrity was analyzed qualitatively and quantitatively by Evans blue infiltration assay. All data were determined by one−way ANOVA and expressed as means ± SEM. **** *p* < 0.0001, ns *p* > 0.05.

Then, we explored the effects of melatonin on the BBB integrity during meningitis TW-XM infection. To test this, the BBB permeability was evaluated by Evans blue infiltration assay, qualitatively and quantitatively, at 12 h post infection, after pretreatment with melatonin for seven consecutive days. As shown in Figure 2A,B, EB could not infiltrate the brain because of its complete BBB in the NS and MT groups. In addition, the brain picture in the TW-XM group showed a deep blue color after EB treatment, suggesting that TW-XM infection severely disrupted the integrity of the BBB with increased permeability, while in the TW-XM + MT group, melatonin administration significantly attenuated the increased permeability of the BBB when compared with the TW-XM group (*p* < 0.001). Meanwhile, there was no statistically significant difference between the NS group and TW-XM + MT group (*p* > 0.05) (Figure 2A,B). These data suggest that the pretreatment of melatonin protects the BBB integrity after APEC TW-XM infection.

### 2.3. Melatonin Supplementation Inhibits the Inflammatory Response and Infiltration of Neutrophils in APEC TW-XM-Infected Mice

**Figure 3:**
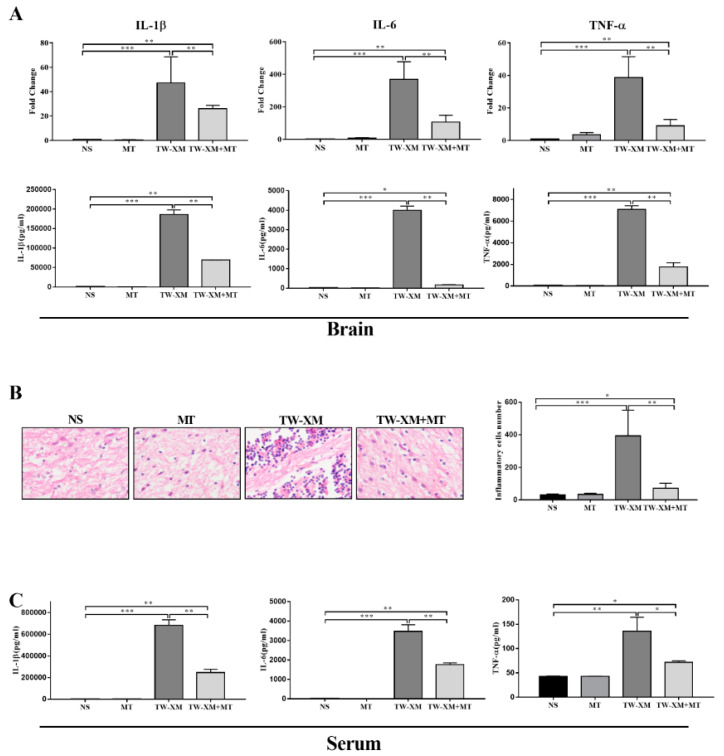
The effect of melatonin on the inflammatory response of brain and serum and the infiltration of neutrophils in the brain in APEC TW−XM−infected mice. (**A**) The mRNA expression and production of inflammatory factors in brain tissues of mice in each group by qPCR. (**B**) The infiltration of neutrophils in the brain was analyzed qualitatively and quantitatively by hematoxylin–eosin staining (bar = 100 μm). (**C**) The production of inflammatory factors in serum of mice in each group by ELISA. All data were determined by one-way ANOVA and expressed as means ± SEM. * *p* < 0.05, ** *p* < 0.01, *** *p* < 0.001.

As shown in Figure 3A, the qPCR results showed that the mRNA expressions of IL-1β, IL-6, and TNF-α in the brain tissue of mice in the TW-XM group and the TW-XM + MT group were significantly higher than those in the NS group (*p* < 0 01). ELISA data further validate the results of qPCR, indicating that APEC TW-XM infection can cause severe inflammation in the brain of mice. However, the expression of IL-1β, IL-6, and TNF-α in the brain tissue of mice in the TW-XM + MT group was significantly lower than that in the TW-XM group, indicating that melatonin can significantly reduce the excessive inflammatory response caused by APEC TW-XM infection and exert an anti-inflammatory effect.

As shown in Figure 3B, a large number of neutrophils infiltrated the brain tissue of mice in the TW-XM group compared to the brain tissue sections of mice in the NS group and the MT group, while the number of neutrophils in the brain tissue of mice in the TW-XM + MT group was significantly less than that in the TW-XM group. The results showed that melatonin could significantly alleviate the brain infiltration of a large number of neutrophils in APEC TW-XM-infected mice.

ELISA results showed the levels of IL-1β, IL-6, and TNF-α in the serum of mice. As shown in Figure 3C, the production of IL-1β, IL-6, and TNF-α in the serum of the TW-XM group and the TW-XM+MT group was significantly higher than that in the NS group and the MT group. Similarly, the production of IL-1β, IL-6, and TNF-α in the serum of the TW-XM+MT group was significantly lower than that in the serum of the TW-XM group. The above results show that melatonin can significantly reduce the systemic inflammation caused by APEC TW-XM infection in mice.

### 2.4. Melatonin Supplementation Maintains Intestinal Microbiota in APEC TW-XM-Infected Mice

**Figure 4:**
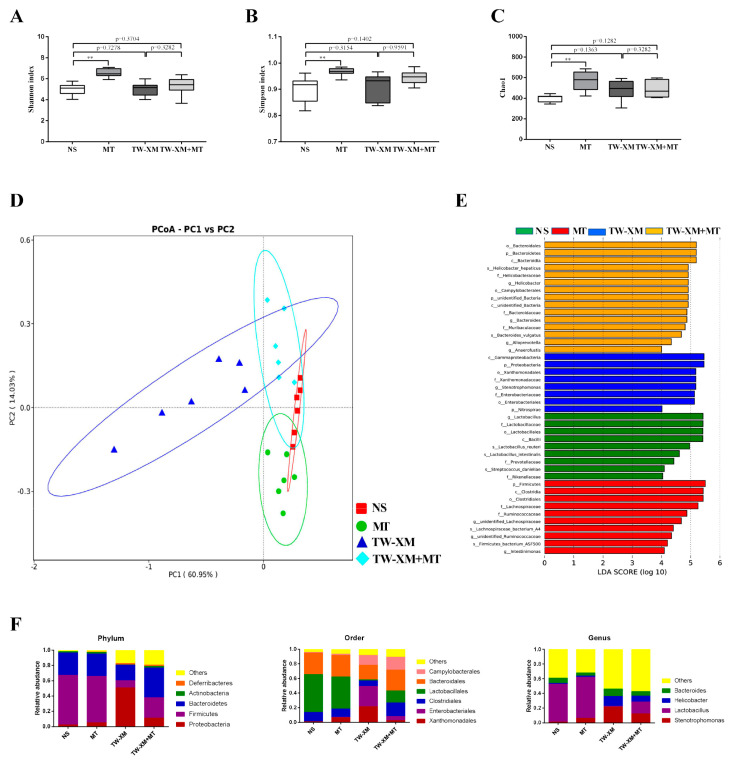
Melatonin maintained intestinal microbiota homeostasis in APEC TW−XM−infected mice. (**A**) Shannon index in α−diversity analysis. (**B**) Simpson index in α−diversity analysis. (**C**) Chao1 in α-diversity analysis. All data were determined by one−way ANOVA and expressed as means ± SEM. ** *p* < 0.01. (**D**) PCoA plot analysis from each sample. (**E**) The taxa whose abundance differed in each group obtained from LEfSe sequence analysis. The cutoff value of ≥4.0 used for the linear discriminant analysis (LDA) is shown. (**F**) The microbiota compositions at the phylum, order, and genus levels.

It has been suggested that the TW-XM infection affected intestinal microbiota in the above clinical symptoms of bacterial meningitis. The effects of melatonin on the intestinal microbiota composition were evaluated by sequencing the 16S rRNA V3 + V4 region. As shown in Figure 4A, the TW-XM group and the TW-XM+MT group had similar α-diversity of colonic intestinal microbiota compared with the NS group, but the α-diversity of colonic intestinal microbiota in the MT group was significantly higher than that in the NS group. Similarly, Simpson (see Figure 4B) quantitatively described the biodiversity of a region and reflected the diversity of the intestinal microbial community. The α-diversity of the NS group was similar to that of the TW-XM group and the TW-XM + MT group, while the α-diversity of the MT group was significantly higher than that of the other three groups. The abundance of Chao1 (see Figure 4C) was consistent with the diversity of Shannon, Simpson, indicating that melatonin pretreatment could increase the diversity of intestinal microorganisms in mice, while APEC TW-XM infection did not significantly affect the diversity of intestinal microbiota.

According to the results of PCoA (Figure 4D), clusters of four groups can be found, that is, the points of each group are concentrated in their respective regions. Compared with the NS group, the MT group is the closest, followed by the TW-XM+MT group and the TW-XM group. It can be seen that APEC TW-XM infection can affect the species composition of intestinal microbiota, and melatonin can prevent the changes in intestinal microbiota species composition induced by the APEC TW-XM infection.

In order to determine the specific bacterial taxa related to melatonin, the differences in intestinal microbiota of mice in the NS group, MT group, TW-XM group, and TW-XM + MT group were compared using the linear discriminant analysis (LDA) effect size (LEfSe) method. The distribution histogram in each group was used to directly analyze the species with significant differences in abundance among the three groups. In addition, the length of the histogram represents the abundance of species with significant differences. As shown in Figure 4E, there were 9 species with significant differences in the NS group, 10 species with significant differences in the MT group, 8 species with significant differences in the TW-XM group, and 15 species with significant differences in the TW-XM+MT group.

Then, we further analyzed the relative abundance of species in Figure 4F. At the phylum level, *Firmicute* had the highest relative abundance of intestinal microbial structures in the NS group and the MT group. In contrast, the relative abundance of *Firmicute* decreased significantly and *Proteobacteria* had the highest abundance of intestinal microbial structures in the TW-XM group. Compared with the TW-XM group, melatonin pretreatment could elevate the relative abundance of *Firmicute* and reduce the relative abundance of *Proteobacteria* in the TW-XM+MT group. At the order level, *Lactobacillales*, *Clostridiales*, and *Bacterioidales* are the dominant strains of NS and MT. Although the relative abundance of *Xanthomonadales* in the intestinal microbiota of the MT group was relatively high compared with the NS group, it was still lower than that of the TW-XM group. In the TW-XM group, *Xanthomonadales*, *Enterobacteriales*, and *Bacterioidales* were the dominant strains in the group. In the TW-XM+MT group, melatonin pretreatment could significantly reduce the relative abundance of *Xanthomonadales* and *Enterobacteriales* and increase the relative abundance of *Clostridiales* and *Lactobacillales* compared with the TW-XM group. At the genus level, *Lactobacillus* was the major dominant strain in the NS group, MT group, and TW-XM + MT group, and *Stenotrophomonas*, *Helicobacter*, and *Bacteroides* were the major dominant strains in the TW-XM group. In the TW-XM+MT group, melatonin pretreatment could significantly reduce the relative abundance of *Stenotrophomonas*, *Helicobacter*, and *Bacteroides*, and increase the relative abundance of *Lactobacillus* compared to the TW-XM group. These results suggest that APEC TW-XM infection can induce changes in the species composition and relative abundance of intestinal microbiota in mice, while melatonin can prevent and improve changes in the species and relative abundance of intestinal microbiota induced by bacteria.

### 2.5. Microbiota Depletion by Antibiotic Block the Anti-Infection Effects of Melatonin in APEC TW-XM-Infected Mice

The above studies have proved that melatonin can prevent meningitis caused by APEC TW-XM infections in mice and found that melatonin can also protect intestinal microbiota homeostasis in meningitis mice. As such, it was speculated that intestinal microbiota may be involved in the potential mechanism of melatonin in preventing bacterial meningitis. Mice were supplemented with normal saline, melatonin, and melatonin+antibiotics for 1 week.

**Figure 5:**
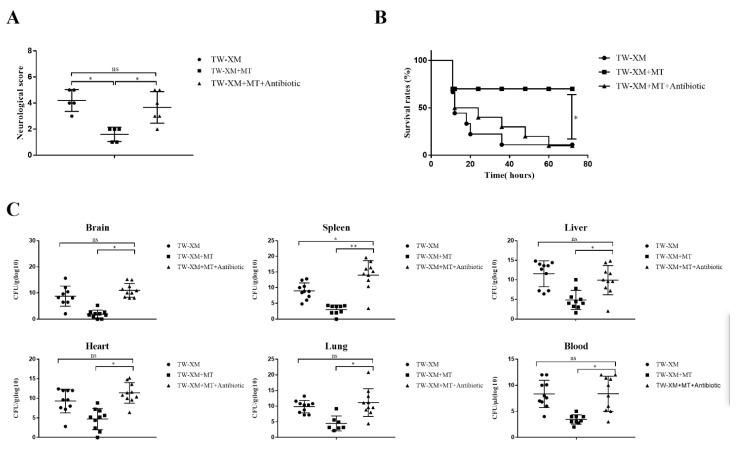
In the absence of intestinal microbiota, melatonin failed to alleviate neurological symptoms, increase survival rates, and reduce bacterial counts of tissues and blood in TW−XM−infected ICR mice. (**A**) The neurological symptom scores of ICR mice in different groups were analyzed at 12 h postinfection. (**B**) The survival rates of ICR mice in different groups were analyzed. (**C**) The loads of TW−XM in brain, spleen, liver, heart, lungs, and blood were analyzed in the TW−XM group and the TW−XM + MT group at 12 h postinfection. Each point represents one mouse. All data were determined by one-way ANOVA and expressed as means ± SEM. * *p* < 0.05, ** *p* < 0.01, ns *p* > 0.05.

Based on the symptom-scoring criteria of meningitis, the symptoms of the APEC TW-XM and TW-XM + MT + Antibiotic groups were compared one week later to evaluate whether melatonin alleviated the symptoms of meningitis by mediating intestinal microbiota in mice. As shown in Figure 5A, the symptoms in the TW-XM + MT group were significantly slighter than those in the TW-XM group, while the severity of the symptoms in the TW-XM + MT + Antibiotic group was significantly higher than that in the TW-XM + MT group (*p* < 0.05). There was no significant difference in meningitis symptoms between the TW-XM + MT + Antibiotic group and the TW-XM group: most of the mice showed lethargy, anorexia, increased eye discharge, and unformed feces at about 12 h. Then, the mice developed convulsions, neck stiffness, and died after frequent angular arch reversal. To sum up, melatonin needs the participation of intestinal microbiota to relieve the neurological symptoms of meningitis in APEC TW-XM-infected mice.

The death onset time of the three groups was about 12–14 h in Figure 5B. In the following time, the survival rates of mice in the TW-XM group and the TW-XM + MT + Antibiotic group decreased. At about 72 h, the final survival rate of mice in both groups was 10%, and that of the TW-XM+MT group was 70%. The above results suggest that intestinal microbiota is involved in melatonin improving the survival rate of APEC TW-XM-infected mice.

At 12 h after intraperitoneal injection of APEC TW-XM, the tissues and blood samples of mice were collected to detect the amounts of bacteria. As shown in Figure 5C, there was no significant difference in the number of APEC TW-XM colonization in the brain, spleen, liver, heart, lungs, or blood between the TW-XM group and the TW-XM + MT + Antibiotic group. Compared with the TW-XM + MT group, the bacterial load in the brain, spleen, liver, heart, lungs, and blood in the TW-XM + MT + Antibiotic group increased significantly. These results suggest that intestinal microbiota participated in melatonin reducing the colonization of APEC TW-XM in various tissues and blood of mice.

### 2.6. Microbiota Depletion by Antibiotic Block the Protection of the Integrity of the BBB from Melatonin in APEC TW-XM-Infected Mice

**Figure 6:**
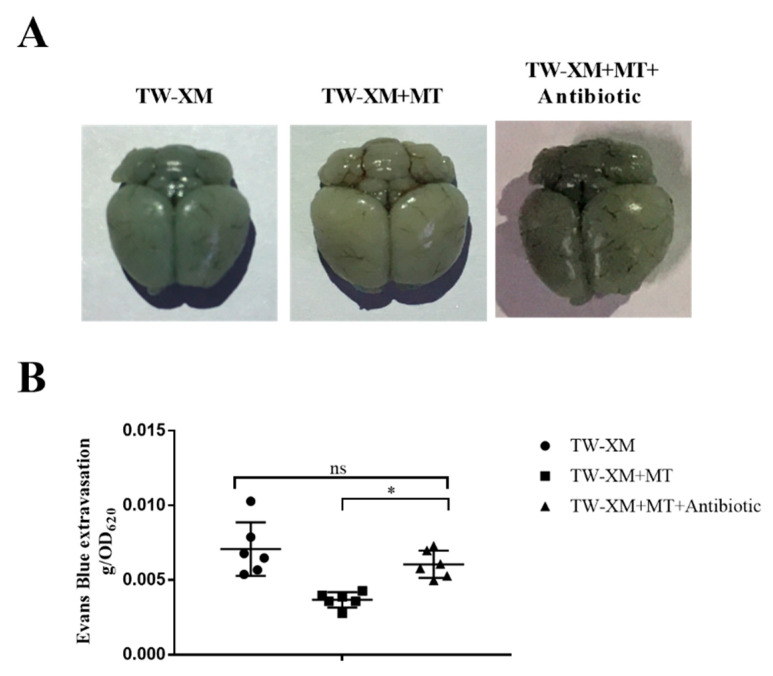
In the absence of intestinal microbiota, melatonin failed to protect the BBB integrity in APEC TW−XM−infected mice. (**A**,**B**) The BBB integrity, analyzed qualitatively and quantitatively by Evans blue infiltration assay. All data were determined by one−way ANOVA and expressed as means ± SEM. * *p* < 0.05, ns *p* > 0.05.

The EB staining of the brain of the three groups of mice is shown in Figure 6A. The brains of the TW-XM group and the TW-XM + MT + Antibiotic group were stained blue and there was no significant difference in EB content, indicating that the integrity of the BBB was damaged. The degree of staining and EB content in the brain of the TW-XM+MT group was significantly lighter than that of the TW-XM group and the TW-XM + MT + Antibiotic group. These results suggest that melatonin can reduce the increase in BBB permeability induced by APEC TW-XM infection in mice, depending on intestinal microbiota.

### 2.7. Microbiota Depletion by Antibiotic Block the Effects of Melatonin on Inhibiting the Inflammatory Response and Infiltration of Neutrophils in APEC TW-XM-Infected Mice

**Figure 7:**
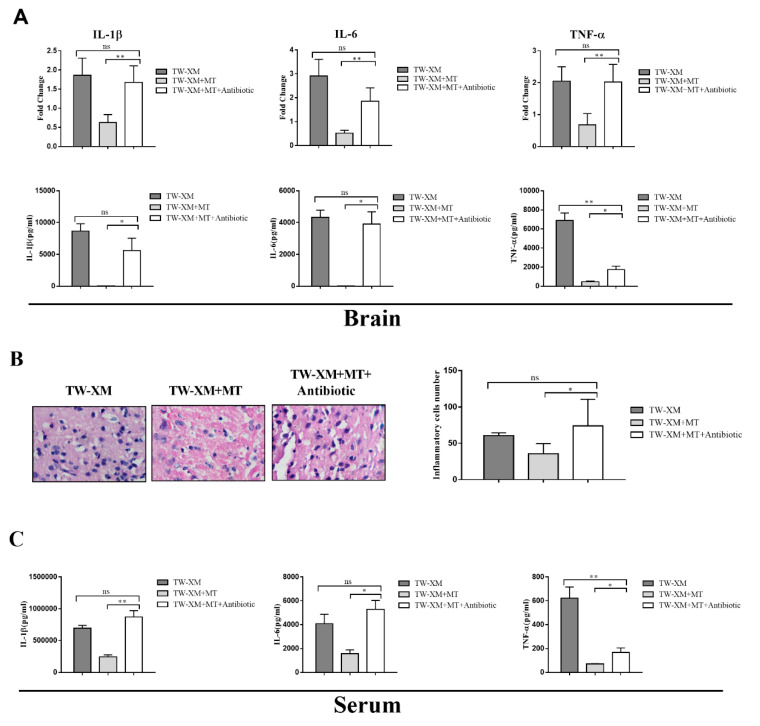
In the absence of intestinal microbiota, melatonin failed to inhibit the inflammatory response of the brain and serum and the infiltration of neutrophils in the brain in APEC TW−XM−infected mice. (**A**) The mRNA expression and production of inflammatory factors in brain tissues of mice in each group by qPCR. (**B**) The infiltration of neutrophils in the brain by hematoxylin–eosin staining (bar=100μm). (**C**) The production of inflammatory factors in serum of mice in each group by ELISA. All data were determined by one-way ANOVA and expressed as means ± SEM. * *p* < 0.05, ** *p* < 0.01, ns *p* > 0.05.

As shown in Figure 7A, there was no significant difference in the relative expression of IL-1β, IL-6, and TNF-α between the TW-XM group and the TW-XM + MT + Antibiotic group, but the relative expression of IL-1β, IL-6, and TNF-α in the TW-XM + MT + Antibiotic group was significantly higher than that in the TW-XM+MT group. The results of ELISA detection in Figure 7A showed that the expression levels of IL-1β and TNF-α in the TW-XM + MT + Antibiotic group were significantly higher than those in the TW-XM group. There was no significant difference in IL-6, but the expression levels of IL-1β, IL-6, and TNF-α were significantly higher than those in the TW-XM+MT group (*p* < 0.001).

We analyzed the pathological changes of HE in the brains of mice. As shown in Figure 7B, there were a large number of neutrophil infiltrations in the brains in the TW-XM + MT + Antibiotic group compared with the TW-XM group, and the neutrophil infiltration in the TW-XM + MT + Antibiotic group was significantly lower than that of the TW-XM + MT group. Therefore, the above results suggest that melatonin inhibits a large number of neutrophil infiltrations, requiring the involvement of intestinal microbiota in the brain of APEC TW-XM-infected mice.

The expression of inflammatory factors in serum was detected, as shown in Figure 7C. There was no significant difference in the expression of IL-1β and IL-6 in the brain tissue of mice in the TW-XM group and the TW-XM + MT + Antibiotic group, but the expression of TNF-α was significantly decreased in the TW-XM + MT + Antibiotic group compared with the TW-XM group. The expression levels of IL-1β, IL-6, and TNF-α in the brain tissue of the TW-XM + MT + Antibiotic group were significantly increased compared with the TW-XM+MT group (*p* < 0.05). It can be seen that melatonin can reduce the expression of inflammatory factors caused by APEC TW-XM infection in mice, depending on intestinal microbiota.

## 3. Discussion

Intestinal microbiota is closely correlated with bacterial meningitis [5,21]. The pathogenic mechanism of *Escherichia coli* meningitis is complex, and bacteremia caused by bacterial invasion of blood, destruction of the blood–brain barrier (BBB), and excessive inflammation are the main factors of meningitis and brain injury [22,23]. Many studies have demonstrated the protective effects of melatonin on nerve injury, the integrity of the BBB, and anti-inflammation in bacterial meningitis [24,25], but its effects on intestinal microbiota had not been explored. In this experiment, we illustrated that melatonin intraperitoneal administration for one week could significantly prevent the occurrence of bacterial meningitis, protect the integrity of the BBB, reduce the bacterial loads in tissues and blood, and alleviate systemic inflammation. Importantly, melatonin was found to maintain the composition of intestinal microbiota, for which the changes induced were closely related to APEC TW-XM infection.

In the meningitis-infected APEC TW-XM mice model, the pretreatment of 30 mg/kg and 60 mg/kg melatonin significantly alleviates the clinical symptoms and improves the survival rate of bacterial meningitis. This is similar to concentrations of melatonin in previous studies for relieving weaning stress and effectively enhances the survival rates of mice infected with *P. multocida* [15,20]. In *K. pneumoniae* meningitis, the neuroprotective treatment effect of melatonin was dose-dependent [12], yet the pretreatment effects of 30 mg/kg and 60 mg/kg melatonin were not dose-dependent in the study. Hence, a melatonin dose of 30 mg/kg as the concentration was to prevent the occurrence and development of meningitis. The amount of bacteria in the tissues and blood in a bacterial infection disease represent systemic infection and bacteremia. After 12 h of APEC TW-XM infection, the amount of bacteria in the blood of mice reached 10^5^ cfu/mL [26], which belonged to high bacteremia. The results show that melatonin pretreatment significantly decreases the amount of bacteria in the blood in APEC TW-XM-infected mice. Although some mice reached the lowest threshold of bacteremia (10^3^ cfu/mL), most of the bacteria carrying capacity of the TW-XM + MT group was lower than the lowest threshold of bacteremia. Similarly, melatonin pretreatment significantly reduced the bacterial load in brain tissue, indicating that melatonin reduced the bacterial load in blood and affected the ability of bacteria to invade the brain tissue. In addition, the same results were found in the spleen, liver, heart, and lungs. Pretreatment of melatonin could significantly reduce the colonization ability of bacteria in various organs. This is consistent with the role of melatonin in preventing the reduction in enterotoxigenic *Escherichia coli* colonization in the intestines of weaned mice [15] and decreasing bacterial loads in the lungs of mice infected with *P. multocida* [20].

The BBB protects the central nervous system and limits the invasion of exogenous pathogens, peripheral cells, and substances [27,28]. When exogenous pathogens invade the BBB and enter the central nervous system, they can induce inflammation in brain, accompanied by an increase in the permeability of the BBB. *Escherichia coli* K1 invasion and destruction of the BBB is an important sign to accelerate the incidence of *Escherichia coli* meningitis and brain injury [29,30]. Our laboratory has studied how the OmpA protein of APEC TW-XM promoted bacterial invasion of the BBB and destroyed the integrity of the BBB (unpublished data). We found that pretreatment of melatonin significantly ameliorated the BBB damage to protect the integrity of the BBB in the APEC TW-XM-infected mice model. In previous studies, systemic inflammation induced by *Escherichia coli* K1 was found to also increase the permeability of the BBB, of which IL-1β, TNF-α, and IL-6 participate in increasing the permeability of the BBB [30,31]. The combined action of TNF-α and IL-6 can directly damage the permeability of brain microvascular endothelial cells, which are the main component of the BBB, and then destroy the BBB. Our studies have shown that melatonin can significantly reduce systemic inflammation and the expression of IL-1β, TNF-α, and IL-6 in the brain of infected mice, thus protecting the integrity of the BBB. In addition, inflammatory factors recruit leukocyte (including neutrophils, monocytes, B cells, and T cells) into the brain for the purpose of removing pathogenic bacteria, but can cause an excessive inflammatory response and lead to severe brain injury and even death [32,33]. In the present study, melatonin pretreatment significantly alleviated massive infiltration of neutrophils in the brain, which suggests that melatonin attenuated the inflammatory response in the brain.

Intestinal microbiota is mainly composed of bacteria, with the highest density living in the colon, which promotes the normal growth of the host by ingesting nutrients and regulates immunity [34,35,36]. In addition, some studies have indicated that intestinal microbiota play a vital role in modulating the inflammatory response of the host. In the healthy intestinal microbiota, *Firmicutes* and *Bacteroides* are usually the dominant microbiota in mammals, and the changes in the composition of intestinal microbiota are different due to different diseases. Therefore, there are some differences in the criteria for judging the imbalance of intestinal microbiota. It has been proved that the infection by *Escherichia coli* K1 can induce the destruction of intestinal homeostasis [37,38], causing changes in the composition of intestinal microbiota. In this paper, the 16S rRNA sequencing technique was used to analyze the intestinal microbiota of mice, and it was found that melatonin pretreatment significantly increased the abundance of *Firmicute* and decreased the abundance of *Proteobacteria* in APEC TW-XM-infected mice, which coincided with the results of the increase in the abundance of *Proteobacteria*, a marker of microbiota imbalance in the previous study [39,40]. This result shows that APEC TW-XM infection can induce intestinal dysbiosis and that melatonin pretreatment can maintain intestinal balance. At the genus level, the abundance of *Stenotrophomonas* in the intestinal microbiota in APEC TW-XM-infected mice was significantly higher than that of the NS group. *Stenotrophomonas* is an opportunistic pathogen, which can cause meningitis, bacteremia, and septicemia [41,42]. Thus, the increase in the proportion of *Stenotrophomonas* could enhance the destruction of intestinal homeostasis and contribute to the occurrence and development of meningitis induced by APEC TW-XM infection in mice, which may result in host inflammation. *Lactobacillus*, as beneficial bacteria, are mainly involved in intestinal immune regulation, promoting the stability of the intestinal environment [43,44]. APEC TW-XM infection can significantly reduce the proportion of *Lactobacillus* in the intestinal tract, leading to reduced intestinal immunity and indicating that APEC TW-XM infection can inhibit the survival and replication of beneficial bacteria. Melatonin has been found to play an important role in the imbalance of microbiota in mice in the study of insomnia and obesity [45,46,47,48]. Melatonin can improve intestinal microbiota, that is, increase the abundance of *Lactobacillus*. In this study, melatonin was found to reduce the decrease in the abundance of beneficial bacteria and the increase in the abundance of harmful bacteria in intestinal microbiota induced by APEC TW-XM and play a protective role in maintaining the balance of intestinal microbiota. However, when mice were pretreated with four kinds of antibiotics to eliminate intestinal microbiota, melatonin pretreatment could not effectively alleviate the clinical symptoms of meningitis, and the survival rate was only 10%. It is suggested that intestinal microbiota are involved in the preventive effect of melatonin on APEC TW-XM-induced meningitis. This result is consistent with the fact that the destruction of microbiota by broad-spectrum antibiotics weakens the host’s resistance to invasive pathogen colonization [49,50]. Melatonin does not effectively reduce the colonization of enterotoxigenic *Escherichia coli* in the intestines of antibiotic-treated weaned mice [15], indicating that melatonin mediates intestinal microbiota to prevent host resistance to pathogen colonization. In addition, previous experimental studies have shown that intestinal microbes and their metabolites play an important role in the formation of the BBB in aseptic mice. Compared with animals fed routinely, the permeability of the BBB by macromolecules in aseptic mice increased significantly [51,52], indicating that intestinal microbiota are involved in the integrity of the BBB. The results show that the integrity of the BBB in mice in the MT + Antibiotics group was destructed compared with the TW-XM+MT group, indicating that melatonin protected the integrity of the blood–brain barrier of meningitis mice by maintain healthy intestinal microbiota. Similarly, it was found that melatonin reduced the expression of inflammatory factors IL-1β, TNF-α, and IL-6 in serum and brain tissue and inhibited a large number of neutrophil infiltrations in brain tissue, depending on healthy intestinal microbiota.

## 4. Materials and Methods

### 4.1. Bacterial Strains

This study used an APEC TW-XM (wild-type, O_2_: H_7_: K_1_), which was isolated from the cerebrospinal fluid of Muscovy ducks with meningitis [53]. APEC TW-XM (TW-XM) was cultured in Luria Bertani (LB) broth medium at 37 °C.

### 4.2. Mice

ICR male mice (3 weeks of age) were purchased from the Comparative Medical Center of Yangzhou University (Yangzhou, China); these mice were maintained in a sterile Trexler-type isolator. The mice were housed in a pathogen-free mouse colony (temperature, 25 ± 2 ℃; relative humidity, 45–60%; lighting cycle, 12 h/d; 7:00–19:00 for light) and had free access to food and drinking water.

### 4.3. Melatonin Supplementation for Weanling Mice

Three-week-old ICR male mice (without receiving any solid food before the experiment) were divided randomly into six groups (*n* = 20/group): the NS (normal saline) group, the MT group, the TW-XM group, and the TW-XM + MT group. The mice in the TW-XM + MT group were intraperitoneally injected at a dosage of 10 mg/kg, 30 mg/kg, and 60 mg/kg in the experimental group for 7 consecutive days before TW-XM infection. In addition, the mice in the MT group and TW-XM + MT group were intraperitoneally injected at a dosage of 30 mg/kg. Meanwhile, the mice in the NS group and the TW-XM group received equal amounts of solvent NS by intraperitoneal injection. The dosage of melatonin used was selected based on previous studies [12,15,19,20]. After one week of MT supplementation, mice of the TW-XM group and the TW-XM + MT group were infected with APEC TW-XM (1.0 × 10^7^ CFUs) by intraperitoneal injection, whereas mice of the solvent NS group and the MT group received equal amounts of NS. The survival rates and neurological symptoms of mice for 5 days were monitored and recorded. Meanwhile, all mice were sacrificed (at 08:00 h) to collect blood samples, brain, heart, lungs, spleen, and liver, which were homogenized aseptically for bacterial counts at 12 h postinfection. The weights of mice were continuously monitored every day during the treatment period. Melatonin (M5250) was purchased from Sigma-Aldrich (St. Louis, MO, USA).

### 4.4. Melatonin Supplementation for Antibiotic-Treated Weanling Mice

Three-week-old ICR male mice (without receiving any solid food before the experiment) were divided randomly into four groups (n = 20/group): the TW-XM group, the TW-XM + MT group, and the TW-XM + MT + Antibiotic group. The mice in the TW-XM + MT group and the TW-XM + MT+ Antibiotic group were intraperitoneally injected at a dosage of 30 mg/kg in the experimental group for 7 consecutive days before TW-XM infection. The mice in the TW-XM group were received equal amounts of solvent NS by intraperitoneal injection. Meanwhile, the mice in the TW-XM group and the TW-XM + MT group received a basal diet and normal drinking fluid, and the mice in the TW-XM + MT+ Antibiotic group received a basal diet and drinking fluid containing streptomycin (1 g/L, Sigma, St. Louis, MO, USA), ampicillin (1 g/L, Sigma), gentamicin (1 g/L, Sigma), and vancomycin (0.5 g/L, Sigma) to clear intestinal bacteria for 7 consecutive days before TW-XM infection. The dosages of melatonin and antibiotics were selected based on previous studies [15]. After one week of MT supplementation and antibiotics treatment, all mice were infected with APEC TW-XM (1.0 × 10^7^ CFUs) by intraperitoneal injection. The survival rates and neurological symptoms of mice for 5 days were monitored and recorded. Meanwhile, all mice were sacrificed (at 08:00 h) to collect blood samples, brain, heart, lungs, spleen, and liver, which were homogenized aseptically for bacterial counts at 12 h postinfection. Meanwhile, the colon and feces were collected for further analyses. The weights of mice were continuously monitored every day during the treatment period.

### 4.5. Tissue Histological Examination

This was performed using hematoxylin and eosin (HE) staining. Briefly, mice brains and colons were fixed with 4% paraformaldehyde–PBS overnight, and then dehydrated and embedded in paraffin blocks. Sections of 10 mg were cut for histological analysis. The sections were deparaffinized and hydrated, and then stained with HE. Then, the tissue sections were analyzed and photographed through optical microscope (Olympus BX51, Tokyo, Japan) with a digital camera. The measurement of neutrophils from each mouse was measured using Image software (Image J, Maryland, USA) [54].

### 4.6. Quantitative Reverse Transcription Polymerase Chain Reaction (qRT-PCR)

Total RNA was extracted and purified from each sample using RNAiso Plus Kit (TIANGEN, Beijing, China) following the manufacturer’s instruction. Purified RNA (2 μg) was used as a template for cDNA synthesis, which was performed using FastKing gDNA Dispelling RT SuperMix (TIANGEN, Beijing, China) following the manufacturer’s instructions. qPCR was performed to determine transcription levels of genes using Faststart Universal SYBR GREEN Master (Roche, Basle, Switzerland) and gene specific primers with 0.5 mg of total RNA. RNA levels were normalized using the housekeeping gene GAPDH transcript and the relative fold change was calculated by using the threshold cycle (△△CT) method. Primers were selected according to previous references (Supplementary Table S1). GAPDH was used as an internal control to normalize target gene transcript levels. qRT PCR was performed according to our previous studies [55].

### 4.7. ELISA

Serum levels of IL-1β, IL-6, and TNF-α, as well as brain levels of IL-1 β, IL-6, and TNF-α were measured using ELISA kits in accordance with the manufacturer’s instructions. The kits were from Cusabio Biotech Company Limited (Wuhan, China). Briefly, supplied diluent buffer in the kits was used to dilute standards and serum samples. Next, 100 μL of the sample or standard in duplicate were added to the wells of a microtiter plate precoated with antibody. Diluent buffer was used as a negative control. The plates were incubated for 2 h at 37 °C. After incubation, 100 μL of biotin antibody was added to each well after removing the liquid and incubated for 1 h at 37 °C. The wells were washed three times with 200 μL volume of wash buffer. Next, 100 μL horseradish peroxidase–avidin was added to each well for 1 h at 37 °C. After a final wash, 90 μL of the supplied TMB substrate was added and incubated for 30 min in the dark at 37 °C. The reaction was stopped with 50 μL of the supplied stop solution and absorbance was measured at 450 nm with a spectrophotometer. The detection ranges of ELISA kits were for IL-1β, IL-6, and TNF-α (SIGA). The coefficients of variation within an assay and between assays were CV% < 9%, and CV% < 10%, respectively. The coefficient of determination of the standard curve was more than 0.95.

### 4.8. Counting of Bacteria

Blood samples, brain, heart, lungs, spleen, liver, intestinal tissues, and feces were homogenized and weighed in PBS and then serial-diluted and plated on MacConkey agar for APEC TW-XM. Bacteria were determined after 16 h of growth at 37 °C. The counts were further verified by colony PCR with specific primers.

### 4.9. Evans Blue

The BBB permeability was assessed by measuring Evans blue (Sigma-Aldrich, St. Louis, MO, USA) extravasations using the modified method of a previous study [39]. Briefly, Evans blue dye (2% in 0.9% saline, 2 mL/kg) was injected into the tail vein immediately at 9 h postinfection. After 3 h, the animals (*n* = 6 per group) were anesthetized with sodium pentobarbital and transcranial perfused with physiological saline, and then decapitated. The brains were removed, and each hemisphere was weighed, homogenized in PBS, and centrifuged at 2000× *g* for 15 min at 4 °C. Then, 0.5 mL of the resulting supernatant was added to an equal volume of trichloroacetic acid. After overnight incubation and centrifugation at 2000× *g* for 15 min at 4 °C, the supernatant was taken for spectrophotometric quantification of extravagated Evans blue dye at 620 nm. The quantitative calculation of the dye content in the brain was based on external standards dissolved in the same solvent. The results are expressed as micrograms per gram of brain tissue.

### 4.10. Gut Microbiota Profiling

Total-genome DNA from fecal samples in the colon was extracted for amplification using specific primer with the barcode (16S V3 + V4). Paired-end sequencing was performed on the Illumina Miseq platform. A phylogenic tree and OUT table were obtained from the mothur Bayesian classifier. Sequencing libraries were generated and analyzed according to Yin’s study [56,57]. Principal coordinated analysis (PCoA) was used to obtain the principal coordinates and visualized from complex, multidimensional data. The complexity of species diversity was evaluated by using Observed-species, Shannon, Simpson, Chao1, and ACE.

### 4.11. Statistical Analysis

All statistical analyses were performed by using the one-way analysis of variance (ANOVA) to test the homogeneity of variances via Levene’s test and followed with the Student’s *t* test (Prism 6.0). Data are expressed as the mean ± SEM. A *p* value of <0.05 was considered significant.

## 5. Conclusions

This study demonstrated that melatonin is a potent preventive agent against APEC TW-XM-induced mice meningitis, decreasing the incidence of bacterial meningitis. The preventive effects of melatonin on the integrity of the BBB, reduced bacterial load in various tissues and blood, and inhibited inflammation and neutrophil infiltration of brain tissue may be dependent on intestinal microbiota. These findings are helpful to further explore the specific mechanism of melatonin-mediated intestinal microbiota in the prevention of and protection against *Escherichia coli* meningitis.

## Data Availability

The sponsor had no role in the study design, data collection, analysis, or interpretation of the data.

## Supplementary Materials

The supporting information can be downloaded at: https://www.mdpi.com/article/10.3390/ijms24010298/s1.
