# Supplementary material for: Melatonin Is Neuroprotective in Escherichia coli Meningitis Depending on Intestinal Microbiota
Source: Int J Mol Sci. 2022 Dec 24;24(1):298. doi: 10.3390/ijms24010298 (PMC9820133; doi:10.3390/ijms24010298)

Supplementary Material

List of materials included: Supplemental Table S1 and Supplemental Figures S1 and S2

Supplementary Table

Supplementary Table S1: List of oligo sequences for qRT-PCR used in the study.

Primers	Sequence (5'-3')	References
IL-6-F	TGCAAGAGACTTCCATCCAGT	[51]
IL-6-R	GTGAAGTAGGGAAGGCCG	[51]
IL-1β-F	ATGAAAGACGGCACACCCAC	[51]
IL-1β-R	GCTTGTGCTCTGCTTGTGAG	[51]
TNF-α-F	AGGCACTCCCCCAAAAGAT	[51]
TNF-α-R	TGAGGGTCTGGGCCATAGAA	[51]
GAPDH-F	ACAACTCACTCAAGATTGTCAGCA	[51]
GAPDH-R	ATGGCATGGACTGTGGTCAT	[51]

Supplementary Figures

Supplementary Figure S1: Symptoms and score of mice in each group after infection. All data were determined by one-way ANOVA and expressed as means ±SEM. $*p < 0.05$, ns $p>0.05$.

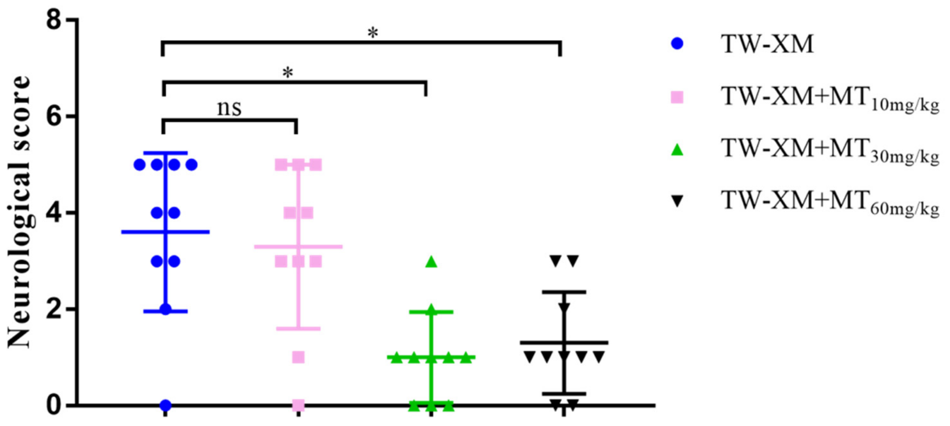

Supplementary Figure S2: The survival rate of mice in each group after APEC TW-XM infection. Each point represents for one mouse. All data were determined by one-way ANOVA and expressed as means ±SEM. *$p < 0.05$, ns $p > 0.05$.

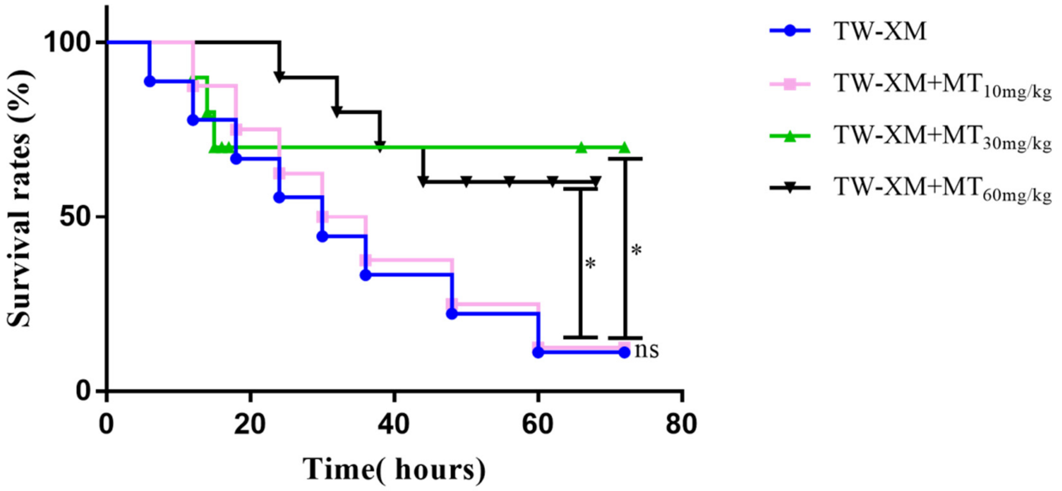

Supplement: Supplementary file 1 [file ijms-24-00298-s001.zip › ijms-2095702-supplementary.pdf]
